# 4535 Development of a Mouse Model to study interactions between parental history of alcohol use and early life adversity on behavioral and neurobiological development of offspring

**DOI:** 10.1017/cts.2020.61

**Published:** 2020-07-29

**Authors:** Grace Porter, Juan Morales, Roslyn Valdespino, Ashley Acheson, Jason O’Connor

**Affiliations:** 1 University of Texas Health Science Center San Antonio

## Abstract

OBJECTIVES/GOALS: Individuals with a family history of alcoholism (FH+) are more likely to develop an alcohol use disorder than those with no such history. Early life adversity has a high coincidence with FH+ making pathogenic studies difficult in clinical studies. Here, we developed a mouse model to study pathogenic mechanisms underlying these risk factors. METHODS/STUDY POPULATION: Male and female C57BL6/J mice were exposed to increasing concentrations of ethanol (3-21%) or water for 15 days prior to breeding. Ethanol was not present during gestation. Offspring were either removed from the home cage and isolated for 3 hours or left undisturbed from postnatal days 1-21. Beginning on PND 56 offspring mice were assessed for clinically relevant behavioral disruptions in social behavior, cognitive working memory, locomotor activity, anxiety-like phenotypes, ethanol preference and binge drinking behavior. In a separate experiment, brains of Cx3cr2^+/GFP^xCcr2^+/RFP^ mice from ELA or control conditions were collected every 7 days after birth for assessment of neuroinflammation and central immune cell morphology and density. RESULTS/ANTICIPATED RESULTS: Mice with a family history of ethanol exposure and ELA are predicted to exhibit behavioral changes (impaired working memory, reduced social behavior, increased anxiety-like behaviors, increased ethanol consumption) to a greater extent than mice with a family history of ethanol exposure or ELA alone. We expect markers of neuroinflammation (cytokine expression, immune cell activation) to predict the behavioral changes in these mice. DISCUSSION/SIGNIFICANCE OF IMPACT: Alcohol consumption and stressful life events are known environmental precipitants to neuroinflammation, which in turns may predispose individuals to anti-social and risky behavior. A mouse model of these early postnatal conditions will allow basic scientists to unravel the biological underpinnings of the behaviors driven by these factors.

